# Risk factors for the development of autism spectrum disorder in children with tuberous sclerosis complex: protocol for a systematic review

**DOI:** 10.1186/s13643-017-0448-0

**Published:** 2017-03-08

**Authors:** Rebecca Mitchell, Sarah Barton, A. Simon Harvey, Katrina Williams

**Affiliations:** 1 Developmental Medicine, The Royal Children’s Hospital, 50 Flemington Road, Parkville, Victoria 3052 Australia; 2 Department of Neurology, The Royal Children’s Hospital, 50 Flemington Road, Parkville, Victoria 3052 Australia

**Keywords:** Tuberous sclerosis complex, Autism spectrum disorder, Children, Seizures, Tubers, White matter disorders, Genotype, Systematic review, Risk factors

## Abstract

**Background:**

Tuberous sclerosis complex (TSC) is an autosomal dominant condition, caused by mutations in either the TSC1 or TSC2 gene. It has widespread systemic manifestations and is associated with significant neurological morbidity. In addition to seizures and cerebral pathology including cortical tubers, subependymal nodules, subependymal giant cell astrocytoma and abnormal white matter, there are recognised neuropsychiatric difficulties including intellectual disability, autism spectrum disorder (ASD) and a range of learning and behaviour problems, recently conceptualised as “tuberous sclerosis-associated neuropsychiatric disorders”, or “TAND”. ASD in TSC is of particular importance because (1) it affects up to 50% of people with TSC and is a source of considerable difficulty for them and their families and (2) it provides a model for considering neurobiological pathways involved in ASD. Multiple factors are implicated in the development of ASD in TSC, including (1) seizures and related electrophysiological factors, (2) cerebral pathology, (3) genotype and (4) child characteristics. However, the neurobiological pathway remains unclear. We will conduct a systematic review to investigate and synthesise existing evidence about the role of these risk factors, individually and in combination, in leading to the development of ASD.

**Methods:**

Our review will report on all studies that include one or more of four predefined risk factors in the development of ASD in children with TSC. We will search five databases: MEDLINE, EMBASE, PubMed, The Cochrane Library and Web of Science (Conference Proceedings Citation Index). Studies will be selected for reporting after two authors independently (1) review all titles and abstracts, (2) read full text of all appropriate papers and (3) assess for bias using the Newcastle-Ottawa Scale recommended by the Guidelines for Meta-Analysis and Systematic Reviews of Observational Studies (MOOSE guidelines) and the ROBINS-I.

**Discussion:**

To our knowledge, this is the first systematic review investigating multiple risk factors in the development of ASD in children with TSC. Clarifying the evidence in this area will be important to researchers in the field and to clinicians providing prognostic information to families.

**Systematic review registration:**

PROSPERO CRD42016042841

**Electronic supplementary material:**

The online version of this article (doi:10.1186/s13643-017-0448-0) contains supplementary material, which is available to authorized users.

## Background

Tuberous sclerosis complex (TSC) is an autosomal dominant condition caused by a mutation in either the TSC1 gene, located on chromosome 9q34.13, or the TSC2 gene, located on chromosome 16p13.3 [1, 2]. In 10–15%, a mutation is not identified. The genes code for the proteins hamartin (TSC1) and tuberin (TSC2) which form a complex that regulates the mTOR cell signalling pathway, responsible for important aspects of cell growth and differentiation [3].

The clinical manifestations of TSC are highly variable. A diagnosis of TSC is made in individuals who meet internationally recognised diagnostic criteria. A definite diagnosis is made when an individual has two or one major with two or more minor clinical features. TSC may also be diagnosed with genetic testing [4].

The major sources of morbidity in TSC are the neurological abnormalities. Epilepsy affects up to 93% of people with TSC [5]. In the brain, cortical tubers, subependymal nodules (SENs), subependymal giant cell astrocytomas (SEGAs) and abnormal white matter are associated with neurodevelopmental problems [6, 7]. Intellectual disability, ASD and other learning and behaviour disorders cause considerable difficulties for children and their families. These problems have recently been conceptualised as “TSC-associated neuropsychiatric conditions” or “TAND” [8].

This review focuses on the relationship between TSC and ASD. Whilst the association between TSC and ASD has been recognised since 1979 [9], it is only since the early 1990s that research in the field has tried to establish why this association exists. ASD is reported to affect between 16 and 55% of children with TSC [10–12]. Understanding the development of ASD in TSC not only is important for individuals affected by TSC but also could provide valuable insights into the development of idiopathic ASD.

The risk factor groups broadly implicated in the neurobiological pathway to ASD are (1) seizures and related electrophysiological factors, (2) neurostructural factors and (3) genotype. Although there has been less of a focus in the literature, the authors also regard (four) individual child characteristics (gender, perinatal history and socio-economic status) and family history of neurodevelopmental problems to be of importance, as these are considered to be central in the etiology of all developmental disorders in children.

Seizures are thought to be pivotal in the development of ASD in TSC. Research has particularly implicated infantile spasms and other seizures that occur at an early age [13–15]. However, this is only part of the picture. Other seizure types, age of seizure onset, seizure burden and interictal EEG activity are all potential contributors. Bombardieri has shown that early control of seizures improves long-term outcomes in children with TSC, demonstrating that children whose seizures were treated within a week of onset did not develop ASD, compared to a group with delayed treatment [16]. Cusmai had similar findings, showing much lower, but not absent, rates of ASD in children whose seizures were treated early [14]. Although Bombardieri’s sample had the TSC2 genotype, neither of these studies accounted for any other co-existent risk factors in analysis. Numis, in a study of children and adults, has considered multiple risk factors simultaneously and found not only that earlier age of onset and more frequent seizures increased ASD risk but also that ASD may be associated with persistent EEG activity in particular brain regions [11]. Which of the seizure factors is of most significance and how these factors interact to affect outcomes in children is unclear.

There is evidence supporting the role of cortical tubers [17–19], SEGAs [20] and white matter disorganisation [21] in ASD development. In early work, Bolton found that the number and temporal lobe location of tubers were associated with ASD [17]. These findings have not been consistently replicated. In contrast, Walz reported tuber location to be of no significance to ASD outcome [22]. Other works have looked at the impact of tuber count on development generally, but not ASD specifically [23, 24]. Furthermore, both Gallagher and Numis have reported that different tuber “types” may impact ASD severity [11, 19].

The presence of the TSC2 gene mutation may also confer a worse overall developmental prognosis [19, 25]. Numis has reported that patients with TSC and ASD had fewer TSC1 mutations [11]. By comparison, Kothare, reporting on a large sample from a natural history database of 919 patients, could not establish a relationship between TSC2 and ASD, but his group acknowledged that data was collected from multiple institutions, with no consistent guidelines for reporting or diagnosing ASD [25].

Whilst there are several reviews of this topic, none has been conducted in a systematic fashion to report on all the published literature. Curatolo’s 2010 review considered pathogenic pathways leading to ASD in TSC, but selected studies based on the novelty and author determined importance and relevance [26]. No reviews have considered the relationship between all four major risk factor groups. This interaction between the risk factors is important, and a review that aims to clarify the contributing role of each factor is needed. In addition, the early life onset of TSC and the developmental problems that arise also mean that a paediatric focus is required. Our review has been methodically designed to report on all paediatric studies that consider a set of predefined risk factors, either separately or in combination, for the development of ASD in TSC.

The risk factors we will consider in our review are as follows:

1. Seizures and related electrophysiological factors.
2. Neurostructural factors (tubers, SEGAs, SENs, white matter abnormalities).
3. TSC genotype.
4. Child characteristics (gender, perinatal factors, socio-economic status and family history of ASD).

A systematic review of the literature to explore what is currently known about the role of risk factors, individually and in combination, is needed to determine future research directions and provide information for better clinical counselling and management.

We aim to answer the questions (1) what are the risk factors for the development of ASD in children with TSC and (2) how do those risk factors interact?

This review is registered on the PROSPERO database (CRD42016042841). Reporting will follow the PRISMA guidelines.

## Methods

### Study eligibility criteria

Cohort studies that consider the impact of any of the predefined risk factors on the development of ASD and case-control studies that report on children with TSC and ASD and investigate associated predefined risk factors will be included.

Studies must specifically address the question of the impact of at least one of the predefined risk factors on the development of ASD in TSC in a paediatric population. The mean age of subjects should be less than 18 years.

The participants for our review are considered to be children (below 18 years of age) with TSC.

The exposure is one or more of the four predefined risk factors for both cohort and case-control studies. For the purposes of this review, the risk factor groups are being considered in the following way:

1. Seizures and related electrophysiological factors: we will report on studies that consider (1) seizure type (and include infantile spasms as a separate subset), (2) age at seizure onset and (3) seizure burden and/or seizure severity. We will report on studies that consider EEG abnormalities including (1) epileptiform discharges and (2) slowing of background activity.
2. Neurostructural factors: we will report on studies that consider the role of (1) tuber number, (2) tuber size, (3) tuber type and (4) total tuber volume. We will report on studies that consider (1) white matter bands or (2) abnormal white matter. We will report on studies that consider SENs or SEGAs (1) number, (2) size or (3) location.
3. Genotype: we will report on studies that consider the role of a TSC1, TSC2 or “unknown TSC” gene mutation.
4. Child characteristics: we will report on studies that consider the role of perinatal factors, child gender, family history of ASD and socio-economic factors in the development of ASD in TSC.

In cohort studies, the comparator is an absence or reduction in exposure to one of the predefined risk factors. In case-control studies, the comparator is children with TSC who do not have ASD.

The primary outcome measure is the incidence of ASD in cohort studies and the presence of any of the four predefined risk factors in case-control studies.

Our exclusion criteria are review papers (systematic or narrative), randomised controlled trials, case reports of less than five children, opinions, letters and editorials. Relevant reviews will have reference lists hand checked.

### Search strategy

Five databases will be searched: MEDLINE, EMBASE, PubMed, The Cochrane Library and Web of Science (Conference Proceedings Citation Index). The database search strategies were developed with the assistance of a senior tertiary hospital librarian with experience in systematic reviews. The strategy was developed to capture all studies that would meet the above eligibility criteria. In addition, the hand searching of review paper references will provide further assurance that all eligible papers have been located. The full search strategy is included as an additional file (Additional file 1).

The search will not be limited to English. When non-English papers are found, journals and authors will be contacted to ask if an English version is available. When an English version is not available, the abstracts will be translated using the Google Translator tool to assess potential eligibility of the paper. If a non-English paper is to be included in the systematic review, we will source translators from our international research networks.

The search will be limited to humans. We are not intending to search for unpublished materials.

### Study selection

The titles and abstracts of all studies generated through the combined database searches will be merged using reference management software and duplicates removed.

All titles and abstracts will be screened by two authors independently. All studies that meet the eligibility criteria on screening titles and abstracts will be sourced and read in full. Where data are not available about subject age, authors will be contacted to provide this, to determine if the study can be included. Disagreements will be resolved through a third author.

A PRISMA study flow diagram will be presented.

### Data extraction

A standardised, pre-piloted form will be used to extract data from the included studies for assessment of quality and evidence synthesis. Extracted information will include (1) number of participants; (2) source of participants (community sample, general hospital sample, tertiary referral centre); (3) participant demographics (age, gender); (4) how the diagnosis of TSC was confirmed (guidelines, clinical notes, other); (5) what ASD diagnostic tools were used; (6) the number of participants with a diagnosis of ASD; (7) what risk factors were assessed (electrophysiological factors, neuroanatomic factors, genotype and child characteristics); (8) how the risk factor exposure was measured; (9) co-occurrence of intellectual disability; (10) reported outcomes, statistical adjustments and statistical significance and (11) information for assessment of risk of bias.

Of importance is the approach that study authors take to the diagnosis of ASD. We intend to report on the method used for diagnosis. We recognise that some studies will diagnose subjects with ASD using a clearly defined diagnostic process or a tool administered by an appropriate person, with wide clinical acceptance. This approach recognises that while multidisciplinary clinical team assessment is the best practice for ASD diagnosis, some tools have acceptable accuracy for diagnosis for research. As such, some studies will determine the presence or absence of ASD using the best practice clinical assessment and apply an appropriate classification system at the time the study was done, with DSM criteria (III/IV/V) or (ICD-9/10). In accordance with the current DSM-V recommendations, studies that have classified children as “autistic disorder”, “Asperger’s disorder” or “pervasive developmental disorder not otherwise specified” will be given the diagnosis of autism spectrum disorder. Other studies may use the Autism Diagnostic Observation Scale (any version) or Autism Diagnostic Interview (original or revised) to make a diagnosis.

### Risk of bias assessment

Risk of bias will be assessed using two tools. We will follow the Guidelines for Meta-Analysis and Systematic Reviews of Observational Studies (MOOSE guidelines) [27], using the Newcastle-Ottawa scale for case-control studies [28] and ROBINS-I for cohort studies [29].

### Data analysis and synthesis

We will enter data into a series of 2 × 2 tables showing ASD or no ASD and exposure to defined risk factor or no exposure to defined risk factor. We will record all relative risk or odds ratios where provided, and will otherwise use the 2 × 2 tables to calculate these values. Confidence intervals, *p* values and any statistical adjustments will be recorded. The decision to report relative risk (RR) or odds ratio (OR) values will be made on the basis of which has been reported in the majority of studies included in that subgroup analysis. We will convert values to the appropriate form for studies using the alternative method.

Data synthesis will be considered separately for each of the four risk factor groups, with an additional section given to discussing any papers that considered the interactions of any of the predefined risk factors. We do not intend to use individual subject data from studies. When pooling results for single-factor studies, we expect that a crude OR will be reported and we will also include crude ORs from multifactor studies into crude OR meta-analyses if this data is available for single factors. We will also conduct meta-analyses of adjusted ORs if more than one study reports adjusted ORs for one or more factors. When adjustments are made for analyses that include single or multiple risk factors, we will extract the factors adjusted for in the analysis and present this information and consider whether it is likely to have influenced reported results. We will pool only studies in which the exposure of interest was measured using similar methods across the pooled studies.

Aggregate subject data will be used and a narrative synthesis presenting quantitative estimates of increased likelihood of a diagnosis of ASD given different risk factors will be done. We plan to present the findings as a series of forest plots for the different risk factors being considered. If the included studies are sufficiently homogenous, a meta-analysis will be considered using a random effects model.

We will consider subgroup analyses to identify important differences in groups when considering (1) source of participants (community sample, general hospital sample, tertiary referral centre), (2) participant demographics (age, gender), (3) how the diagnosis of TSC was confirmed (guidelines, clinical notes, other), (4) what ASD diagnostic tools were used, (5) how the risk factor exposure was measured, (6) co-occurrence of intellectual disability and (7) overall risk of bias.

We will assess heterogeneity by visually inspecting forest plots and calculating *I* ^2^ statistic before pooling data. If there is high heterogeneity, we will assess whether clinical or methodological factors have contributed to this, and if there are clinical features that have led to the heterogeneity, we will perform and present subgroup analyses, so the cause and magnitude of differences created by clinical variation are clear.

To assess the potential impact of methodological differences, we will perform sensitivity analyses, when sufficient studies using different methods are available, and report the effect of the inclusion and exclusion of different methods.

We will assess for publication bias. Whilst there are no specific guidelines for assessing this in systematic reviews of cohort studies, our approach will be to consider adjusted and unadjusted risk factor effects separately and observe any asymmetry in results. These results will then determine the appropriateness of further statistical testing.

### Overall quality of evidence (GRADE system)

We plan to give an overall assessment of the quality of the body of evidence using the GRADE approach [30].

The methodology for this review was developed using the PRISMA-P checklist (Additional file 2).

## Discussion

The review aims to report on the likelihood of the development of ASD in a child with TSC exposed to one or more of the risk factors being studied. Firstly, this provides valuable prognostic information for families and allows clinicians to flag children likely to need referral to early intervention services. Secondly, it assists in identifying pathways that contribute to ASD development, providing opportunities to target medical interventions. Finally, clarifying what the literature currently states about the role of clinical risk factors in the development of ASD in TSC is important for streamlining and directing future research efforts.

## Amendments to the protocol

The protocol published with PROSPERO had undergone one amendment prior to the submission of this manuscript for publication. The amendment was to include Web of Science (Conference Proceedings Citation Index) in the database search. Any future amendments to the protocol will also be registered with PROSPERO and available online.

## Dissemination

Our intention is to submit the review to a major international journal in the field for publication.

## Additional files

Additional file 1: The full search strategy. (PDF 636 kb)
Additional file 2: PRISMA-P checklist. (DOC 85 kb)

## Abbreviations

ASD: Autism spectrum disorder
SEGA: Subependymal giant cell astrocytoma
SEN: Sub ependymal nodule
TAND: Tuberous sclerosis-associated neuropsychiatric disorders
TSC: Tuberous sclerosis complex
