# Supplementary material for: Risk factors for the development of autism spectrum disorder in children with tuberous sclerosis complex: protocol for a systematic review
Source: Syst Rev. 2017 Mar 8;6:49. doi: 10.1186/s13643-017-0448-0 (PMC5341363; doi:10.1186/s13643-017-0448-0)
Supplement: Additional file 1: — The full search strategy. (PDF 636 kb) [file 13643_2017_448_MOESM1_ESM.pdf]

Cochrane

Search Name: Risk Factors July

Last Saved: 15/07/2016 02:32:11.311

Description:

ID Search

#1 MeSH descriptor: [Tuberous Sclerosis] explode all trees

#2 tubero* near/1 sclerosis (Word variations have been searched)

#3 MeSH descriptor: [Child Development Disorders, Pervasive] explode all trees

#4 (development* next disorder*) or "developmental status" or (disintegrative next disorder*) or pervasive or autism or autistic or asperger* (Word variations have been searched)

#5 MeSH descriptor: [Risk Factors] this term only

#6 MeSH descriptor: [Risk] this term only

#7 MeSH descriptor: [Genotype] this term only

#8 MeSH descriptor: [Phenotype] this term only

#9 MeSH descriptor: [Epilepsy] this term only

#10 MeSH descriptor: [Astrocytoma] this term only

#11 MeSH descriptor: [Magnetic Resonance Imaging] this term only

#12 MeSH descriptor: [Diffusion Magnetic Resonance Imaging] this term only

#13 MeSH descriptor: [Electroencephalography] this term only

#14 MeSH descriptor: [TOR Serine-Threonine Kinases] this term only

#15 MeSH descriptor: [Socioeconomic Factors] this term only

#16 MeSH descriptor: [Prenatal Exposure Delayed Effects] this term only

#17 MeSH descriptor: [Pregnancy Outcome] this term only

#18 (prognostic next factor*) or risk* or electrophysiologic* or electro-physiologic* or (electro next physiologic*) or epileps* or epileptic or seizure* or interictal or inter-ictal or (inter next ictal) or electroencephalogra* or electro-encephalogra* or (electro next encephalogra*) or (infant* next spasm*) or astrocytoma or gene or genes or genetic* or genotyp* or phenotyp* or mutation* or tuber or (white next matter) or SEN* or SEGA or (subependymal next nodule*) or gender or famil* or MRI* or neuroimaging or neuro-imaging or (neuro next imaging) or (magnetic next resonance next imaging) or (image next analys*) or socioeconomic* or socio-economic* or (socio next economic*) or (prenatal* near/3 expos*) or (pregnanc* near/3 outcome*) or (target near/3 rapamycin) or serine-threonine or (serine next threonine) or (mtor near/3 protein*) (Word variations have been searched)

#19 gestation* or embryo* or prenatal* or neonat* or newborn* or infan* or toddler* or child* or preschooler* or pre-schooler* or adolescen* or paediatric* or pediatric* (Word variations have been searched)

#20 MeSH descriptor: [Child] explode all trees

#21 MeSH descriptor: [Infant] explode all trees

#22 MeSH descriptor: [Adolescent] explode all trees

#23 (#1 or #2) and (#3 or #4) and (#5 or #6 or #7 or #8 or #9 or #10 or #11 or #12 or #13 or #14 or #15 or #16 or #17 or #18) and (#19 or #20 or #21 or #22)

PubMED

(tuberous sclerosis) AND (“asperger syndrome” OR autism OR “autistic disorder” OR “autism spectrum” OR childhood disintegrative disorder* OR disintegrative disorder* OR pervasive OR “pervasive developmental disorder not otherwise specified” OR child development* disorder* OR development* disorder* OR “developmental status”) AND (“diffusion magnetic resonance imaging” OR “diffusion MRI” OR “diffusion tensor imagining” OR “diffusion weighted imaging” OR electrophysiologic* OR electroencephalography OR epilepsy OR epileptic OR family OR family’s OR

familial OR “family history” OR “functional neuroimaging” OR gene OR genes OR genetic* OR genotyp* OR “gene mutation” OR “genotype phenotype” OR “genotype phenotype correlation” OR gender OR “ image analysis” OR interictal OR inter-ictal OR “magnetic resonance imagining” OR neuroimaging OR “nuclear magnetic resonance imaging” OR “perfusion weighted imaging” OR “prenatal exposure delayed effects” OR “pregnancy outcome” OR prognostic factor* OR risk OR risk factor* OR seizure OR “social class” OR socioeconomic factor* OR “TOR Serine-Threonine Kinases” OR tuberous OR tuber OR “white matter”) AND (embryo* OR prenatal* OR neonat* OR infan* OR toddler* OR child* OR preschooler* OR pre-schooler* OR adolescen* OR paediatric* OR pediatric*) AND (NOTNLM OR publisher[sb] OR inprocess[sb] OR pubmednotmedline[sb] OR indatareview[sb] OR pubstatusaheadofprint)

Database(s): **Ovid MEDLINE(R)** 1946 to July Week 1 2016

Search Strategy:

#	Searches	Results	Annotations
1	Tuberous Sclerosis/	5306	
2	child development disorders, pervasive/ or autism spectrum disorder/ or autistic disorder/ or asperger syndrome/ or ((development* adj disorder*) or (developmental adj status)).tw,kf.	30305	
3	risk/ or risk factors/	755586	
4	genotype/ or phenotype/	341039	
5	Epilepsy/	67110	
6	Astrocytoma/	13762	
7	(gene*1 or genetic* or genotyp* or electrophysiologic* or epilepsy or epileptic or seizure* or interictal or inter-ictal or (infant* adj spasm*) or tuber* or (white adj matter) or SEN* or (subependymal adj nodule*) or SEGA* or (subependymal adj giant adj cell adj astrocytoma*) or gender or famil*).tw,kf,hw.	5422834	
8	magnetic resonance imaging/ or diffusion magnetic resonance imaging/	333706	
9	neuroimaging.tw,kf,hw.	33866	
10	Electroencephalography/	129262	
11	(risk*1 or (prognostic adj factor*1)).tw,kf,hw.	1843859	
12	TOR Serine-Threonine Kinases/	13226	
13	ge.fs.	2848736	
14	Socioeconomic Factors/	129055	
15	prenatal exposure delayed effects/	23334	
16	pregnancy outcome/	41708	
17	(gestation* or embryo* or prenatal* or neonat* or newborn* or infan* or toddler* or child* or preschooler* or pre-schooler* or adolescen* or paediatric* or pediatric*).tw,kf,hw.	3990572	
18	1 and 2 and (3 or 4 or 5 or 6 or 7 or 8 or 9 or 10 or 11 or 12 or 14 or 15 or 16) and 17	113	
19	1 and 2 and 13 and 17	40	
20	18 or 19	113	

1. **Autism spectrum disorder in tuberous sclerosis complex: searching for risk markers.**

Vignoli A; La Briola F; Peron A; Turner K; Vannicola C; Saccani M; Magnaghi E; Scornavacca GF; Canevini MP.

Orphanet Journal Of Rare Diseases. 10:154, 2015.
[Journal Article. Research Support, Non-U.S. Gov't]

UI: 26631248

BACKGROUND: Neuropsychiatric **disorders** are present in up to 90% of patients with **Tuberous** Sclerosis Complex (TSC), and represent an important issue for **families**. Autism Spectrum **Disorder** (ASD) is the most common neurobehavioral disease, affecting up to 61% of patients. The aims of this study were: 1) to assess the prevalence of ASD in a TSC population; 2) to describe the severity of ASD; 3) to identify potential **risk factors** associated with the **development** of ASD in TSC patients.
METHODS: We selected 42 individuals over age 4 years with a definite diagnosis of TSC and followed at a TSC clinic in Northern Italy. We collected and reported clinical and **genetic** data, as well as cognitive level, for each of them. We administered the Social Communication Questionnaire (SCQ) as a reliable screening tool for ASD, and performed comparisons between the average scores and each clinical and **genetic** feature.
RESULTS: Seventeen out of 42 patients (40.5%) had a score at the SCQ suggestive of ASD (>15 points). When calculated for each cognitive level category, the average SCQ score tended to be progressively higher in patients with a worse cognitive level, and the number of pathological SCQ scores increased with worsening of intellectual disability. With respect to ASD severity, the scores were equally distributed, indicating that the degree of

Wolters Kluwer
Ovid®
My Account | Ask a Librarian | Support & Training | VICTORIA | Help | Logged in as Rebecca Mitchell at Royal Children's Hospital Melbourne | Logoff

Search | Journals | Books | Multimedia | My Workspace | OvidMD

Search History (20)

View Saved

# ▲	Searches	Results	Type	Actions	Annotations
1	(gene*1 or genetic* or genotyp* or electrophysiologic* or epilepsy or epileptic or seizure* or interictal or inter-ictal or (infant* adj spasm*) or tuber* or (white adj matter) or SEN* or (subependymal adj nodule*) or SEGA* or (subependymal adj giant adj cell adj astrocytoma*) or gender or famil*).tw,kw,hw.	7332896	Advanced	Display Results | More	
2	(risk*1 or (prognostic adj factor*1)).tw,kw,hw.	2868397	Advanced	Display Results | More	
3	(gestation* or embryo* or prenatal* or neonat* or newborn* or infan* or toddler* or child* or preschooler* or pre-schooler* or adolescen* or paediatric* or pediatric*).tw,kw,hw.	4359842	Advanced	Display Results | More	
4	tuberous sclerosis/	9107	Advanced	Display Results | More	
5	autism/ or asperger syndrome/ or childhood disintegrative disorder/ or "pervasive developmental disorder not otherwise specified"/ or ((development* adj disorder*) or (developmental adj status)).tw,kw.	50483	Advanced	Display Results | More	
6	risk factor/	757269	Advanced	Display Results | More	
7	seizure/	120735	Advanced	Display Results | More	
8	epilepsy/	121063	Advanced	Display Results | More	
9	astrocytoma/	18883	Advanced	Display Results | More	
10	target of rapamycin kinase/	2950	Advanced	Display Results | More	
11	exp gene mutation/	505965	Advanced	Display Results | More	
12	electroencephalogram/	82746	Advanced	Display Results | More	
13	image analysis/	141947	Advanced	Display Results | More	
14	nuclear magnetic resonance imaging/ or diffusion tensor imaging/ or diffusion weighted imaging/ or perfusion weighted imaging/	595685	Advanced	Display Results | More	
15	neuroimaging/ or functional neuroimaging/	91011	Advanced	Display Results | More	
16	genotype phenotype correlation/	13705	Advanced	Display Results | More	
17	Socioeconomics/	121708	Advanced	Display Results | More	
18	prenatal exposure/	19388	Advanced	Display Results | More	
19	pregnancy outcome/	39569	Advanced	Display Results | More	
20	5 and 4 and (1 or 2 or 6 or 7 or 8 or 9 or 10 or 11 or 12 or 13 or 14 or 15 or 16 or 17 or 18 or 19) and 3	369	Advanced	Display Results | More	

Contract

Save | Remove | Combine with: AND | OR

Save All | Create RSS | View Saved

Basic Search | Find Citation | Search Tools | Search Fields | **Advanced Search** | Multi-Field Search

1 Resource selected | Hide | Change

Embase 1974 to 2016 Week 28

Enter keyword or phrase (* or $ for truncation) — Keyword | Author | Title | Journal

Search

Limits *(close)* — Include Multimedia — Map Term to Subject Heading

Full Text | Latest Update | Abstracts
Human | Cochrane Library | English Language

Publication Year - | -

Additional Limits | Edit Limits

To search Open Access content on Ovid, go to Basic Search.

Results Tools
