# Supplementary material for: Risk factors for the development of autism spectrum disorder in children with tuberous sclerosis complex: protocol for a systematic review
Source: Syst Rev. 2017 Mar 8;6:49. doi: 10.1186/s13643-017-0448-0 (PMC5341363; doi:10.1186/s13643-017-0448-0)
Supplement: Additional file 2: — PRISMA-P checklist. (DOC 85 kb) [file 13643_2017_448_MOESM2_ESM.doc]

**Additional file 2**

**PRISMA-P (Preferred Reporting Items for Systematic review and Meta-Analysis Protocols) 2015 checklist: recommended items to address in a systematic review protocol***

| Section and topic | Item No | Checklist item |
| --- | --- | --- |
| ADMINISTRATIVE INFORMATION | | |
| Title: |  |  |
| Identification | 1a | Identify the report as a protocol of a systematic review – Done |
| Update | 1b | If the protocol is for an update of a previous systematic review, identify as such – No it is not. |
| Registration | 2 | If registered, provide the name of the registry (such as PROSPERO) and registration number – PROSPERO CRD42016042841 |
| Authors: |  |  |
| Contact | 3a | Provide name, institutional affiliation, e-mail address of all protocol authors; provide physical mailing address of corresponding author – Done |
| Contributions | 3b | Describe contributions of protocol authors and identify the guarantor of the review – author contributions described. |
| Amendments | 4 | If the protocol represents an amendment of a previously completed or published protocol, identify as such and list changes; otherwise, state plan for documenting important protocol amendments – Done |
| Support: |  |  |
| Sources | 5a | Indicate sources of financial or other support for the review – there is no funding being provided for this systematic review. The first author is undertaking the review as part of her PhD through the University of Melbourne, Australia. Professor Katrina Williams and Dr Simon Harvey, as PhD supervisors, will review and contribute as required. Dr Barton has an appointment with the Department of Neurology at the Royal Children’s Hospital and is taking on the role of contributing author as part of her duties. |
| Sponsor | 5b | Provide name for the review funder and/or sponsor – Not applicable |
| Role of sponsor or funder | 5c | Describe roles of funder(s), sponsor(s), and/or institution(s), if any, in developing the protocol – Not applicable |
| INTRODUCTION | | |
| Rationale | 6 | Describe the rationale for the review in the context of what is already known – Done |
| Objectives | 7 | Provide an explicit statement of the question(s) the review will address with reference to participants, interventions, comparators, and outcomes (PICO) – Done (Intervention=Exposure)  The question for this systematic review is; We aim to answer the questions; (1) what are the risk factors for the development of autism spectrum disorder in children with tuberous sclerosis complex, and (2) how do those risk factors interact?  PI(E)COS:  Participants: Children with a diagnosis of Tuberous Sclerosis Complex (TSC)  Exposure: A set of pre-defined risk factors detailed in the manuscript  Comparator: Children with TSC who do not have an ASD (case-control), Children without exposure/reduced exposure to one or more pre-defined risk factors (cohort studies).  Outcomes: Presence or absence of ASD in children (cohort studies), Presence of any of the pre-defined risk factors (case-control studies) |
| METHODS | | |
| Eligibility criteria | 8 | Specify the study characteristics (such as PICO, study design, setting, time frame) and report characteristics (such as years considered, language, publication status) to be used as criteria for eligibility for the review - Done |
| Information sources | 9 | Describe all intended information sources (such as electronic databases, contact with study authors, trial registers or other grey literature sources) with planned dates of coverage – Done |
| Search strategy | 10 | Present draft of search strategy to be used for at least one electronic database, including planned limits, such that it could be repeated – Done |
| Study records: |  |  |
| Data management | 11a | Describe the mechanism(s) that will be used to manage records and data throughout the review – Done |
| Selection process | 11b | State the process that will be used for selecting studies (such as two independent reviewers) through each phase of the review (that is, screening, eligibility and inclusion in meta-analysis) – Done |
| Data collection process | 11c | Describe planned method of extracting data from reports (such as piloting forms, done independently, in duplicate), any processes for obtaining and confirming data from investigators – Done |
| Data items | 12 | List and define all variables for which data will be sought (such as PICO items, funding sources), any pre-planned data assumptions and simplifications – Done |
| Outcomes and prioritization | 13 | List and define all outcomes for which data will be sought, including prioritization of main and additional outcomes, with rationale – Done |
| Risk of bias in individual studies | 14 | Describe anticipated methods for assessing risk of bias of individual studies, including whether this will be done at the outcome or study level, or both; state how this information will be used in data synthesis – As indicated in the protocol this will be done at individual study level. |
| Data synthesis | 15a | Describe criteria under which study data will be quantitatively synthesised – Done |
|  | 15b | If data are appropriate for quantitative synthesis, describe planned summary measures, methods of handling data and methods of combining data from studies, including any planned exploration of consistency (such as I^2^, Kendall’s τ)- Done |
|  | 15c | Describe any proposed additional analyses (such as sensitivity or subgroup analyses, meta-regression) – Done |
|  | 15d | If quantitative synthesis is not appropriate, describe the type of summary planned – Done |
| Meta-bias(es) | 16 | Specify any planned assessment of meta-bias(es) (such as publication bias across studies, selective reporting within studies) – Publication bias will be considered. |
| Confidence in cumulative evidence | 17 | Describe how the strength of the body of evidence will be assessed (such as GRADE) – We will assess the overall quality of evidence using a GRADE approach. |

*** It is strongly recommended that this checklist be read in conjunction with the PRISMA-P Explanation and Elaboration (cite when available) for important clarification on the items. Amendments to a review protocol should be tracked and dated. The copyright for PRISMA-P (including checklist) is held by the PRISMA-P Group and is distributed under a Creative Commons Attribution Licence 4.0.**

*From: Shamseer L, Moher D, Clarke M, Ghersi D, Liberati A, Petticrew M, Shekelle P, Stewart L, PRISMA-P Group. Preferred reporting items for systematic review and meta-analysis protocols (PRISMA-P) 2015: elaboration and explanation. BMJ. 2015 Jan 2;349(jan02 1):g7647.*
